# Physical Fitness, Experiential Avoidance, and Psychological Inflexibility Among Adolescents: Results from the EHDLA Study

**DOI:** 10.3390/children12081032

**Published:** 2025-08-06

**Authors:** Maria Mendoza-Muñoz, José Francisco López-Gil, Damián Pereira-Payo, Raquel Pastor-Cisneros

**Affiliations:** 1 Department of Communication and Education, Universidad Loyola Andalucía, 41014 Sevilla, Spain; mamendozam@unex.es; 2 School of Medicine, Universidad Espíritu Santo, Samborondón 092301, Ecuador; 3 Vicerrectoría de Investigación y Postgrado, Universidad de Los Lagos, Osorno 5290000, Chile; 4 Health, Economy, Motricity and Education (HEME) Research Group, Faculty of Sport Sciences, University of Extremadura, 10003 Cáceres, Spain; dpereirapayo@unex.es; 5 Physical Activity for Education, Performance and Health Research Group (PAEPH), Faculty of Sport Sciences, University of Extremadura, 10003 Cáceres, Spain; raquelpc@unex.es

**Keywords:** experiential avoidance, mental health, physical activity, physical education, physical fitness, psychological inflexibility

## Abstract

**Highlights:**

**What are the main findings?**

- In unadjusted models, certain components of physical fitness (cardiorespiratory fitness, agility, and flexibility) showed significant associations with psychological inflexibility in adolescents.
- After adjusting for key confounders, none of the physical fitness components maintained a significant relationship with psychological inflexibility.

**What is the implication of the main finding?**

- Physical fitness alone does not appear to be a primary determinant of psychological inflexibility during adolescence.
- Future interventions targeting psychological flexibility may require integrated approaches that go beyond physical conditioning to include psychosocial and emotional components.

**Abstract:**

**Background/Introduction:** Psychological inflexibility, which includes experiential avoidance, is a transdiagnostic process associated with multiple mental health issues in adolescence. Physical fitness (PF) has shown benefits for mental well-being, yet its specific relationship with psychological inflexibility remains understudied, particularly among youth. **Objectives:** To examine the association between components of PF and psychological inflexibility, measured by the Acceptance and Action Questionnaire-II (AAQ-II), in a representative sample of Spanish adolescents. **Methods:** A cross-sectional analysis was conducted using data from 631 adolescents (aged 12–17) participating in the Eating Healthy and Daily Life Activities (EHDLA) study. PF was assessed by the Assessing the Levels of PHysical Activity and Fitness (ALPHA-Fit) Test Battery (cardiorespiratory fitness, muscular strength, agility, and flexibility). Psychological inflexibility was measured using the AAQ-II. Generalized linear models (GLMs) were used to evaluate associations, adjusting for age, sex, body mass index, socioeconomic status, physical activity, sedentary behavior, sleep duration, and energy intake. **Results:** Unadjusted analyses showed weak but significant associations between psychological inflexibility and performance in the 20 m shuttle run test (*p* = 0.002), the 4 × 10 shuttle run test (*p* = 0.005), and the sit-and-reach test (*p* < 0.001). However, after adjusting for covariates, none of the PF components maintained a statistically significant association with the AAQ-II scores. **Conclusions:** In this adolescent sample, PF components were not independently associated with psychological inflexibility after adjustment for key confounders. These findings suggest that, while PF may contribute to general well-being, it is not a primary determinant of psychological inflexibility. Further longitudinal and intervention studies are needed to clarify the mechanisms linking physical and psychological health in youth.

## 1. Introduction

Physical fitness (PF) is a fundamental indicator of health, particularly during childhood and adolescence, as it reflects the body’s ability to perform daily physical tasks efficiently and safely. It comprises multiple components (e.g., muscular strength, muscular endurance, cardiorespiratory endurance, flexibility, and body composition) that together determine an individual’s physical performance capacity. This multidimensional construct has been widely recognized by leading institutions such as the American College of Sports Medicine (ACSM) for its relevance in both clinical and public health contexts [1]. Genetic inheritance determines part of an individual’s PF; however, environmental factors and lifestyle may also have an influence on it, with physical exercise being fundamental in this regard [2]. The various components of PF have been related to several physical and mental health variables. Specifically in children and adolescents, high levels of PF have been inversely related to total and abdominal obesity [3]. Muscle strength and cardiorespiratory fitness (CRF) have also been related to metabolic risk in adolescence [4]. Thus, PF is becoming established as a powerful indicator of health status [5].

Experiential avoidance refers to a psychological process in which individuals attempt to alter the forms, frequencies, or situational triggers of certain internal experiences (e.g., bodily sensations, emotions, thoughts, memories, images, behavioral predispositions, etc.) because they find them aversive or distressing. These efforts to avoid or suppress internal events often persist despite resulting in functional impairment or negative behavioral consequences [6]. The definition of psychological inflexibility by Bond et al. [7] refers to the rigid dominance of psychological reactions over chosen values and contingencies in guiding actions, making the definitions of psychological inflexibility and experiential avoidance very similar [8]. Both constructs have been associated with a range of mental health problems, such as anxiety [9], depression [10], and trauma in adolescents.

Research has shown that PF is positively correlated with emotional well-being, and that regular physical activity (PA) (a key determinant of PF) can reduce symptoms of anxiety and depression [11]. Specifically, in adolescents, CRF was inversely associated with depression [12,13], with lower levels of psychological difficulties [14] and with self-esteem and variables related to physical perception [15,16].

Thus, regular PA is essential for adolescents as it helps develop healthy lifestyle habits and improves mental and physical functioning. In contrast, experiential avoidance was negatively associated with PA [10], hindering the ability to engage in PA, which may perpetuate mental health problems.

These constructs are crucial for understanding and improving adolescent mental health, as psychological inflexibility and experiential avoidance have been linked to various emotional and behavioral issues. However, research in this area is limited, particularly among young populations. Therefore, this study aims to fill this gap by exploring the relationships between psychological flexibility, experiential avoidance, and PF among adolescents, thereby providing a foundation for future interventions and prevention programs.

## 2. Materials and Methods

### 2.1. Study Design and Sample Size

A cross-sectional study was carried out to analyze data from a representative sample of adolescents aged 12–17 years from *Valle de Ricote* (Region of Murcia, Spain) enrolled in the Eating Healthy and Daily Life Activities (EHDLA) [17]. The sample consisted of 603 adolescents (56.1% girls) from three secondary schools. The data were collected during the academic year 2021/2022.

Prior to participation, parents or legal guardians of the adolescents were provided with a written information sheet outlining the aims of the research project, along with detailed descriptions of the assessments and questionnaires involved. They were required to sign an informed consent form before their child could be enrolled in this study. Additionally, adolescents themselves provided assent, indicating their voluntary agreement to take part in the research.

The following inclusion criteria were considered for this study: (1) being between 12 and 17 years old; and (2) being registered and/or residing in the *Valle de Ricote* (Region of Murcia, Spain). In addition, the exclusion criteria were as follows: (1) adolescents who were exempt from the subject of Physical Education at school (since all the measurements in this study were carried out during Physical Education classes); (2) adolescents who suffered from a pathology that prevented them from practicing PA; (3) adolescents who were under some type of pharmacological treatment; (4) adolescents whose parents or legal guardians did not authorize their participation in the study; or (5) adolescents who did not agree to participate in the study.

This study received ethical approval from both the Bioethics Committee of the University of Murcia (ID-2218/2018) and the Ethics Committee of the Albacete University Hospital Complex and Albacete Integrated Care Management (ID-2021-85). All procedures were conducted in accordance with the revised Declaration of Helsinki, ensuring full compliance with international standards for the ethical treatment of human participants and the protection of their rights and well-being.

### 2.2. Measures

#### 2.2.1. Experiential Avoidance and Psychological Inflexibility

Experiential avoidance and psychological inflexibility were assessed using the Acceptance and Action Questionnaire-II (AAQ-II) [7], a widely employed instrument in psychological research. Participants completed the validated Spanish version, which has demonstrated strong psychometric properties, including high internal consistency (Cronbach’s alpha = 0.88) [18]. The AAQ-II comprises seven items, each rated on a 7-point Likert scale, ranging from 1 to 7. The items denote unwillingness to experience unwanted emotions and thoughts (e.g., ‘I am afraid of my feelings’ and ‘I worry that I cannot control my worries and feelings’) and the inability to be in the present moment and behave towards value-directed actions when experiencing psychological events that might undermine them (e.g., ‘My painful experiences and memories make it difficult for me to live a life I would value’, ‘My painful memories prevent me from having a fulfilling life’, and ‘Worries get in the way of my success’).

#### 2.2.2. Physical Fitness

The ALPHA-Fit Test Battery was used to evaluate PF [19,20]. The PF components assessed were lower body strength, upper body strength, CRF, speed agility, and flexibility.

The handgrip strength test using a hand dynamometer (TKK 5041 Grip D, Takei, Tokio, Japan) was used to assess upper body strength. The digital dynamometer was grip-adjustable, and a table of estimated reference values was used to adjust the grip. The absolute handgrip strength (kg) was calculated by averaging the readings from both the left and right hands and subsequently normalized to body weight.

Lower limb strength was assessed using the Standing Broad Jump Test. It was measured in centimeters using a tape measure (from the toe-off line to the back point of the heel) [21].

CRF was estimated as the maximum volume of oxygen consumed during a maximum incremental field test (20 m shuttle run test). During the test, participants continuously ran back and forth between two markers placed 20 m apart, adjusting their pace in accordance with pre-recorded audio signals. The test was finished when the participant either voluntarily stopped due to exhaustion or failed to reach the designated line in synchrony with the audio cue on two consecutive occasions [22,23]. The final completed stage of half-lap was used as the performance score.

Speed agility was measured through the 4 × 10 m shuttle run test [20], which involved sprinting back and forth between two lines set 10 m apart, while retrieving and placing three sponges as quickly as possible to complete a total of 40 m.

Flexibility was measured by the sit-and-reach test [24], performed on a standardized box equipped with a rule mounted on the top (height: 33 cm). With hands aligned and arms extended forward, the participant sat on the floor with both legs fully extended, shoulder-width apart and feet touching the box, and the participant stretched by sliding their hand forward along the ruler and held the final position for at least 2 s. The score was recorded in centimeters.

To calculate a global physical fitness (PF) index, scores from the five individual tests were converted into age- and sex-standardized z-scores using the following formula: z-score = (value − mean)/standard deviation (SD).

Notably, since a longer time spent in the 4 × 10 m shuttle run test (indicating slower speed and agility) implies poorer performance in this component of PF, the obtained z-score value was subsequently reversed (i.e., multiplied by −1) to facilitate the direct comparison among the various PF components. Subsequently, the sum of these z-scores was calculated to establish the overall PF score [25,26].

#### 2.2.3. Covariates

Demographic information, including age and sex, was collected from each participant. Socioeconomic status was assessed using the Family Affluence Scale (FAS-III) [27], which assigns scores ranging from 0 to 13. Higher scores indicate greater affluence. PA levels and sedentary behavior were measured using the Youth Physical Activity Profile (YAP) [28]. The instrument relies on self-report to evaluate a range of PAs and sedentary behaviors undertaken during the previous seven days. Body mass index (BMI) was derived by dividing each participant’s weight (in kilograms) by the square of their height (in meters). Weight was recorded using a digital scale with a precision of 0.1 kg (Tanita BC-545, Tokyo, Japan), while height was measured to the nearest 0.1 cm using a portable stadiometer (Leicester Tanita HR 001, Tokyo, Japan). Sleep duration was estimated by averaging weekday and weekend sleep times.

### 2.3. Statistical Analysis

The distribution of the data was evaluated through quantile–quantile plots, density curves, and the Shapiro–Wilk test to determine normality. Categorical variables presented absolute and relative frequencies, while non-normally distributed continuous variables were described using medians and interquartile ranges (IQRs). As no significant interaction was observed between sex and PF components regarding AAQ-II scores (*p* > 0.05 in all cases), subsequent analyses were performed on the sample. Generalized linear models (GLMs) with robust standard errors (‘SMDM’ method) and assuming a Gaussian distribution were employed to address potential heteroscedasticity and outlier effects. The results are reported as unstandardized beta coefficients (*B*) with 95% confidence intervals (CIs). In addition, adjusted R^2^ values were computed to assess the explanatory power of each model. All models were adjusted for age, sex, socioeconomic status, BMI, sleep duration, PA, sedentary behavior, and energy intake. Statistical analyses were performed using R software (v4.4.0) and RStudio (v2024.04.1+748), with statistical significance set at *p* < 0.05.

## 3. Results

Descriptive results for the adolescent sample are shown in Table 1, which included 603 adolescents with a median age of 14.0 years (56.1% were girls). The median score on FAS-III was 8.0, indicating a relatively high socioeconomic status. Participants reported a median sleep duration of 501.4 min per night and moderate levels of PA (median Youth Activity profile – Spanish version [YAP-S] PA score: 2.6) and sedentary behavior (median YAP-S sedentary behavior score: 2.6). The median daily energy intake was 1681.6 kcal.

Anthropometric data indicate a median BMI of 21.7 kg/m^2^. For PF, the median number of laps completed in the 20 m shuttle run test was 29.0, and the median handgrip strength was 21.9 kg. Performance on the standing long jump test averaged 139.0 cm, while the 4 × 10 shuttle run test had a median completion time of 12.7 s. Flexibility, assessed through the sit-and-reach test, showed a median of 25.0 cm. Z-scores for muscular fitness and overall PF had medians of approximately 0, reflecting normalization within the sample. The median AAQ-II score, indicative of psychological inflexibility, was 22.0 (IQR: 13.0–30.0), suggesting moderate levels of experiential avoidance.

**Figure 1:**
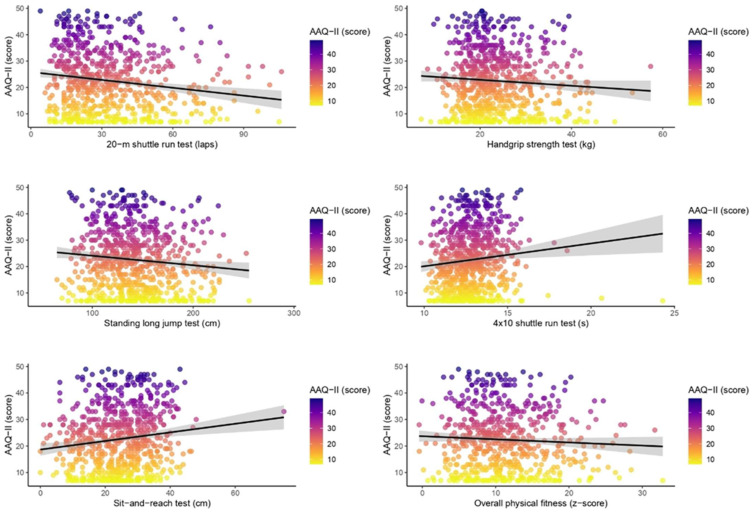
The unadjusted association between the different components of physical fitness and Acceptance and Action Questionnaire-II scores among adolescents. The black line indicates the unadjusted linear regression fit; the gray area represents the 95% confidence interval.

Figure 1 shows scatterplots with regression lines illustrating the associations between various components of PF and psychological inflexibility, as measured by AAQ-II scores. An inverse trend is observed for the 20 m shuttle run, handgrip strength, standing long jump, and overall PF (z-score), suggesting that higher physical performance is associated with slightly lower AAQ-II scores (indicative of greater psychological flexibility). Specifically, in the 4 × 10 m test, longer completion times (reflecting lower performance) are associated with higher AAQ-II scores. For the sit-and-reach test, greater performance is also linked to slightly higher AAQ-II scores. Nevertheless, the regression slopes across all analyses are shallow, and the data are widely dispersed, indicating weak and likely non-significant associations.

Table 2 presents the results of linear regression analyses examining the associations between PF components and the AAQ-II among adolescents. Although the 20 m shuttle run test (*p* = 0.002), the 4 × 10 shuttle run test (*p* = 0.005), and the sit-and-reach test (*p* < 0.001) showed a significant association with psychological inflexibility as measured by the AAQ-II, after adjusting for age, sex, socioeconomic status, PA, sedentary behavior, sleep duration, body mass index, and energy intake, none of the PF variables showed statistically significant associations with psychological inflexibility. The adjusted coefficients were small (*B* from −0.04 to 0.46), and their 95% confidence intervals included zero. These findings suggest that, after accounting for key demographic and behavioral factors, PF levels do not appear to be significantly related to psychological inflexibility in this adolescent population. The full results of all the GLMs are found in the Supplementary Material (Tables S1–S6).

## 4. Discussion

The present study aimed to explore the relationship between PF and psychological inflexibility in a sample of Spanish adolescents. While the initial unadjusted analyses suggested weak associations between certain PF components (CRF, agility, and flexibility) and psychological inflexibility, these relationships did not remain significant after adjusting for relevant sociodemographic, lifestyle, and anthropometric covariates. This finding highlights the potential influence of these covariates, such as sex, socioeconomic status, sedentary behavior, or sleep patterns, on psychological inflexibility as measured by the AAQ-II. These findings provide new evidence for the complex interaction between physical and psychological aspects during adolescence. These findings contrast with prior research indicating that higher levels of PA and PF are related to other desirable psychological outcomes in adolescents, including reduced depressive and anxiety symptoms [29], enhanced emotional regulation [30], and improved self-esteem [31]. For instance, CRF has been identified as a particularly strong predictor of emotional resilience and positive mental health outcomes [11,32]. Moderate physical exercise has also been associated with reduced psychological inflexibility, improved social adaptability, and greater perseverance [33,34]. Shen et al. [35] found that, in adolescents, PA negatively predicted psychological inflexibility, suggesting that those with lower inflexibility are better equipped to manage stress through active coping strategies [36].

However, our results suggest that once important confounders such as socioeconomic status, sleep, energy intake, and PA are considered, PF alone does not independently predict lower levels of psychological inflexibility. This may be partly due to the development of more adaptive emotional regulation strategies, such as acceptance, problem solving, and emotional control, among physically active adolescents, possibly driven by PA’s effects on brain plasticity and executive functioning [37,38]. Conversely, individuals with low fitness levels may rely more heavily on maladaptive strategies, including experiential avoidance [39].

In addition, sex-specific biological factors may influence both physical and psychological responses. For instance, hormonal fluctuations during the menstrual cycle can alter proprioceptive control and neuromuscular performance in adolescent females [40], potentially modifying the relationship between PF and psychological flexibility. Future studies should consider these physiological dynamics when examining sex differences in adolescent populations.

Biological mechanisms may also explain this relationship. PA has been shown to reduce hypothalamic–pituitary–adrenal axis activation and lower cortisol levels, a hormone associated with chronic stress [41]. It also stimulates the release of neurotransmitters such as endorphins, serotonin, and dopamine, which are essential for mood regulation and psychological well-being [42]. Moreover, regular PA contributes to improved emotional control through structural and functional changes in brain areas involved in stress response, such as the amygdala, hippocampus, and prefrontal cortex [41,43]. Additionally, higher levels of PF may increase self-efficacy and perceived control over adverse situations. These two factors are closely linked to adaptive coping strategies. Tang et al. [44] highlighted the mediating role of self-efficacy in the relationship between PA and the reduction in negative emotions, including anxiety and depression. This supports the idea that fitness contributes to a greater sense of competence in managing emotions, which in turn enhances psychological resilience [45]. These mechanisms could help explain why adolescents with higher CRF in our study showed slightly lower experiential avoidance scores, even if the associations were not statistically significant after adjustment.

These mechanisms could help explain why adolescents with higher CRF in our study showed slightly lower experiential avoidance scores, even if the associations were not statistically significant after adjustment. Frequent engagement in PA may lead to a generally more active lifestyle, involving greater exposure to challenging or uncomfortable situations and, consequently, improved tolerance to emotional discomfort. Socially, PA (especially in adolescents) can also foster supportive environments and social skills, which act as protective factors against avoidance-based coping strategies [38,46].

Nevertheless, our adjusted findings suggest that, while PF may contribute to overall mental well-being, it does not appear to be an independent determinant of psychological inflexibility in adolescents. This aligns with previous research indicating that the psychological benefits of PA may be mediated by affective and cognitive variables rather than arising directly from PF. For instance, recent studies have highlighted the role of emotional self-efficacy, perceived control, and social support as mediators in the relationship between PA and mental health outcomes [44,45]. Furthermore, a recent longitudinal anti-doping analysis conducted among elite Italian athletes revealed an increase in the use of antidepressants, anxiolytics, and cannabinoids over the past decade, suggesting that even physically fit and high-performing individuals are not immune to psychological distress [47]. In line with this, it is also plausible that some of the covariates included in our models (e.g., sex, socioeconomic status, sedentary behavior, and sleep duration) may act as mediators or moderators in the association between physical fitness and psychological inflexibility. Indeed, the attenuation of associations observed after adjustment suggests that these variables may partly explain the initial relationships. However, while we did speculate the potential mediators, we did not conduct formal mediation analyses. Therefore, interpretations of these variables as mediators remain hypothetical and should be interpreted with caution. Future studies should incorporate mediation modeling to explore these mechanisms in a greater depth.

On the other hand, Alcaraz-Ibáñez, Sicilia, and Burgueño [34] also found that psychological inflexibility and social physique anxiety were more strongly associated with motivational and emotional factors than with fitness indicators themselves. Similarly, Pakenham et al. [36] reported that psychological flexibility was more closely associated with cognitive–emotional resilience than with physical parameters. These findings help explain why the associations between PF components and experiential avoidance observed in unadjusted models attenuated when controlling for psychosocial and behavioral confounders. Thus, evidence suggests that these psychological approaches yield more specific and sustained effects on experiential avoidance than physical exercise or PF alone [10,34,36]. Therefore, school and community-based programs should consider combining structured PA with brief psychological interventions designed to enhance present-moment awareness, value-based action, and emotional tolerance mechanisms in the development of psychological flexibility during adolescence [8,10,33].

Recent evidence supports the feasibility and effectiveness of brief, low-intensity psychological interventions for adolescents, particularly in improving internalizing symptoms and fostering emotional self-regulation [48]. Integrating these targeted strategies with physical fitness programs may enhance their synergistic potential. In addition, the growing accessibility of digital health platforms offers promising avenues for expanding the reach and personalization of mental health promotion among youth. Technology-based approaches may complement traditional fitness-based interventions by increasing engagement and adaptability in adolescent populations [49].

Although our findings did not support the initial hypothesis, they did provide valuable insights that contributed meaningfully to the existing body of knowledge. Scientifically sound null results are essential in advancing psychological and health sciences, as they help delineate the boundaries within which certain associations do or do not exist. In this case, our results suggest that the relationship between PF and psychological inflexibility in adolescents may not be as direct or robust as previously hypothesized, particularly when adjusting for key sociodemographic and behavioral confounders. This highlights the need for caution in assuming linear or causal links between physical and psychological domains without accounting for contextual complexity. Furthermore, these findings underscore the importance of adopting multifactorial and integrative models that incorporate emotional regulation, cognitive style, family dynamics, social context, and lifestyle behaviors (alongside physical fitness) when exploring psychological processes such as experiential avoidance or inflexibility. Understanding adolescent psychological functioning therefore demands a more holistic approach that moves beyond isolated variables and embraces the complex interplay of biological, psychological, and social determinants of health.

This study is the first to examine PF in relation to psychological inflexibility using a multidimensional fitness battery and validated measures in adolescents. It underscores the importance of examining psychological outcomes through a multilevel lens, accounting for not only physical attributes but also behavioral, social, and cognitive domains. However, certain limitations should be acknowledged. First, the cross-sectional design does not allow for causal conclusions. Second, the use of self-reported data for psychological inflexibility and behavioral covariates may be influenced by recall errors or social desirability bias, particularly relevant in adolescent populations reporting on mental health and lifestyle behaviors. Third, as the sample was drawn exclusively from the Valle de Ricote region (Murcia, Spain), findings may not generalize to adolescents from other cultural, socioeconomic, or geographic backgrounds. Fourth, the effect sizes observed in our models were small, even before adjustment, which suggests that the relationship between and psychological inflexibility may be weak or highly dependent on other variables. Therefore, our findings should be interpreted with caution, especially when considering their practical significance. Fifth, although variables such as sleep duration, sedentary behavior, and socioeconomic status were included as covariates in the adjusted models (allowing a preliminary exploration of their potential mediating roles), we did not perform formal mediation analyses to quantify indirect effects or causal pathways. Therefore, interpretations of these variables as mediators should be made with caution. Sixth, incorporating qualitative methodologies or mixed-methods approaches may provide deeper insight into adolescents’ subjective experiences of stress, avoidance, and resilience. Interventions combining PF with psychological components (e.g., mindfulness, acceptance-based therapies) could also be evaluated for their potential synergistic effects. Lastly, although the final sample size (n = 603) likely provided sufficient power to detect small-to-moderate effects, we did not perform an a priori power analysis based on previously reported associations. This limits the methodological rigor of the study design. Future research should incorporate power estimations derived from the existing literature to guide appropriate sample sizes.

## 5. Conclusions

PF does not appear to be a key determinant of psychological inflexibility in adolescent population. No significant associations were found between PF components and psychological inflexibility, as measured by the AAQ-II, after adjusting for key confounders such as age, sex, socioeconomic status, PA, sedentary behavior, sleep duration, body mass index, and energy intake. Although descriptive analyses and unadjusted models suggested weak trends, such as inverse associations for CRF and muscular fitness and positive trends for agility and flexibility, these relationships did not remain significant in adjusted models. These results highlight the complexity of psychological processes during adolescence and suggest that other psychosocial or environmental factors may play a more central role in shaping experiential avoidance and psychological flexibility.

Further longitudinal and intervention studies are needed to clarify the potential mechanisms linking physical health and psychological functioning in youth and to explore whether specific types or intensities of PA may influence psychological flexibility over time.

## Figures and Tables

**Table 1:** Descriptive data of the study participants.

Variable	*N* = 603
Age (years)	14.0 (13.0, 15.0)
Sex	
Male	265 (43.9%)
Female	338 (56.1%)
FAS-III (score)	8.0 (7.0, 9.0)
Overall sleep duration (minutes)	501.4 (458.6, 531.4)
YAP-S physical activity (score)	2.6 (2.2, 3.1)
YAP-S sedentary behaviors (score)	2.6 (2.2, 3.0)
Energy intake (kcal)	1681.6 (1294.7, 2296.2)
BMI (kg/m^2^)	21.7 (19.3, 25.0)
20 m shuttle run test (laps)	29.0 (20.0, 43.0)
Handgrip strength test (kg)	21.9 (18.6, 27.5)
Standing long jump test (cm)	139.0 (120.0, 161.0)
Muscular fitness (z-score)	−0.1 (−0.7, 0.6)
4 × 10 shuttle run test (s)	12.7 (12.0, 13.5)
Sit-and-reach test (cm)	25.0 (18.0, 31.0)
Overall physical fitness (z-score)	0.0 (−0.7, 0.7)
AAQ-II (score)	22.0 (13.0, 30.0)

Median (interquartile range) or number (percentage). AAQ-II, Acceptance and Action Questionnaire-II; BMI, body mass index; FAS-III, Family Affluence Scale-III; YAP-S, Youth Activity Profile – Spanish version.

**Table 2:** Both unadjusted and adjusted association between physical fitness components and Acceptance and Action Questionnaire-II score among adolescents.

	Unadjusted				Adjusted ^†^			
Predictor	*B*	95% CI	*p*-Value	Adjusted *R*^2^	*B*	95% CI	*p*-Value	Adjusted *R*^2^
20 m shuttle run test (per one lap)	−0.08	−0.14 to −0.03	0.002	0.02	−0.03	−0.09 to 0.03	0.407	0.13
Handgrip strength test (per one kilogram)	−0.10	−0.23 to 0.04	0.155	0.002	−0.01	−0.17 to 0.15	0.902	0.12
Standing long jump test (per one centimeter)	−0.03	−0.06 to 0.004	0.085	0.004	0.002	−0.03 to 0.03	0.907	0.12
4 × 10 shuttle run test (per one second)	1.09	0.33 to 1.84	0.005	0.01	0.46	−0.37 to 1.29	0.275	0.13
Sit-and-reach test (per one centimeter)	0.18	0.08 to 0.28	<0.001	0.02	0.04	−0.07 to 0.14	0.487	0.13
Overall physical fitness (per one SD)	−0.11	−0.28 to 0.07	0.222	0.001	−0.04	−0.22 to 0.15	0.696	0.12

*B*, unstandardized beta coefficient; CI, confidence interval; *R*^2^, coefficient of determination; SD, standard deviation. ^†^ Adjusted for age, sex, socioeconomic status, physical activity, sedentary behavior, sleep duration, body mass index, and energy intake.

## Data Availability

The raw data supporting the conclusions of this article will be made available by the authors upon reasonable request, due to ethical restrictions related to the participation of minors.

## Supplementary Materials

The following supporting information can be downloaded at: https://www.mdpi.com/article/10.3390/children12081032/s1.

## Abbreviations

The following abbreviations are used in this manuscript:

AAQ-II	Acceptance and Action Questionnaire-II
BMI	Body mass index
CI	Confidence interval
CRF	Cardiorespiratory fitness
FAS-III	Family Affluence Scale-III
GLM	Generalized linear model
PA	Physical activity
PF	Physical fitness
R^2^	Coefficient of determination
SD	Standard deviation
YAP	Youth Activity Profile
YAP-S	Youth Activity Profile – Spanish version
