# Supplementary material for: Physical Fitness, Experiential Avoidance, and Psychological Inflexibility Among Adolescents: Results from the EHDLA Study
Source: Children (Basel). 2025 Aug 6;12(8):1032. doi: 10.3390/children12081032 (PMC12384701; doi:10.3390/children12081032)
Supplement: Supplementary file 1 [file children-12-01032-s001.zip › children-3768559-supplementary.pdf]

Supplementary material

Table S1. Unadjusted and adjusted associations between 20-m shuttle run test and experiential avoidance among adolescents.

	Unadjusted model			Adjusted model		
**Predictor**	***B***	**95% CI**	***p*-value**	***B***	**95% CI**	***p*-value**
20-m shuttle run test (per one lap)	-0.08	-0.14, -0.03	0.002	-0.03	-0.09, 0.03	0.407
FAS-III (per one point)				-0.44	-0.88, 0.00	0.049
Age (per one year)				0.28	-0.37, 0.94	0.402
Sex						
Boys				Ref.		
Girls				6.40	4.33, 8.46	<0.001
Energy intake (per 1000 kcal)				0.00	0.00, 0.00	0.180
YAP-S physical activity (per one point)				0.51	-0.90, 1.92	0.478
YAP-S sedentary behaviors (per one point)				1.58	0.01, 3.15	0.050
BMI (per one kg/m^2)				0.12	-0.09, 0.33	0.264
Overall sleep duration (per one hour)				-0.04	-0.05, -0.02	<0.001

B, unstandardized beta coefficient; BMI, body mass index; CI, confidence interval; FAS-III, Family Affluence Scale-III; Ref., reference; YAP-S, Youth Active Profile Spanish version.

Table S2. Unadjusted and adjusted associations between handgrip strength test and experiential avoidance among adolescents.

	Unadjusted model			Adjusted model		
**Predictor**	***B***	**95% CI**	***p*-value**	***B***	**95% CI**	***p*-value**
Handgrip strength (per one kg)	-0.10	-0.23, 0.04	0.155	-0.01	-0.17, 0.15	0.920
FAS-III (per one point)				-0.46	-0.90, -0.03	0.037
Age (per one year)				0.21	-0.48, 0.90	0.551
Sex						
Boys				Ref.		
Girls				6.73	4.62, 8.84	<0.001
Energy intake (per 1000 kcal)				0.00	0.00, 0.00	0.179
YAP-S physical activity (per one point)				0.40	-0.98, 1.78	0.572
YAP-S sedentary behaviors (per one point)				1.62	0.04, 3.19	0.044
BMI (per one kg/m^2)				0.15	-0.05, 0.36	0.138
Overall sleep duration (per one hour)				-0.03	-0.05, -0.02	<0.001

B, unstandardized beta coefficient; BMI, body mass index; CI, confidence interval; FAS-III, Family Affluence Scale-III; Ref., reference; YAP-S, Youth Active Profile Spanish version.

Table S3. Unadjusted and adjusted associations between standing long jump test and experiential avoidance among adolescents.

	Unadjusted model			Adjusted model		
**Predictor**	***B***	**95% CI**	***p*-value**	***B***	**95% CI**	***p*-value**
Standing long jump (per one cm)	-0.03	-0.06, 0.00	0.085	0.00	-0.03, 0.03	0.907
FAS-III (per one point)				-0.47	-0.90, -0.03	0.036
Age (per one year)				0.18	-0.47, 0.84	0.587
Sex						
Boys				Ref.		
Girls				6.83	4.78, 8.87	<0.001
Energy intake (per 1000 kcal)				0.00	0.00, 0.00	0.183
YAP-S physical activity (per one point)				0.39	-0.99, 1.78	0.578
YAP-S sedentary behaviors (per one point)				1.62	0.05, 3.19	0.044
BMI (per one kg/m^2)				0.16	-0.05, 0.36	0.137
Overall sleep duration (per one hour)				-0.03	-0.05, -0.02	<0.001

B, unstandardized beta coefficient; BMI, body mass index; CI, confidence interval; FAS-III, Family Affluence Scale-III; Ref., reference; YAP-S, Youth Active Profile Spanish version.

Table S4. Unadjusted and adjusted associations between 4x10 shuttle run test and experiential avoidance among adolescents.

	Unadjusted model			Adjusted model		
**Predictor**	***B***	**95% CI**	***p*-value**	***B***	**95% CI**	***p*-value**
4x10 shuttle run test (per one s)	1.09	0.33, 1.84	0.005	0.46	-0.37, 1.29	0.275
FAS-III (per one point)				-0.42	-0.86, 0.02	0.059
Age (per one year)				0.31	-0.35, 0.96	0.357
Sex						
Boys				Ref.		
Girls				6.34	4.33, 8.36	<0.001
Energy intake (per 1000 kcal)				0.00	0.00, 0.00	0.163
YAP-S physical activity (per one point)				0.46	-0.92, 1.85	0.511
YAP-S sedentary behaviors (per one point)				1.56	-0.01, 3.14	0.052
BMI (per one kg/m^2)				0.12	-0.09, 0.32	0.255
Overall sleep duration (per one hour)				-0.04	-0.05, -0.02	<0.001

B, unstandardized beta coefficient; BMI, body mass index; CI, confidence interval; FAS-III, Family Affluence Scale-III; Ref., reference; YAP-S, Youth Active Profile Spanish version.

Table S5. Unadjusted and adjusted associations between sit-and-reach test and experiential avoidance among adolescents.

	Unadjusted model			Adjusted model		
**Predictor**	***B***	**95% CI**	***p*-value**	***B***	**95% CI**	***p*-value**
Sit-and-reach test (per one cm)	0.18	0.08, 0.28	<0.001	0.04	-0.07, 0.14	0.487
FAS-III (per one point)				-0.47	-0.90, -0.04	0.034
Age (per one year)				0.18	-0.44, 0.80	0.570
Sex						
Boys				Ref.		
Girls				6.51	4.47, 8.54	<0.001
Energy intake (per 1000 kcal)				0.00	0.00, 0.00	0.163
YAP-S physical activity (per one point)				0.39	-0.99, 1.77	0.581
YAP-S sedentary behaviors (per one point)				1.64	0.07, 3.21	0.041
BMI (per one kg/m^2)				0.15	-0.04, 0.35	0.128
Overall sleep duration (per one hour)				-0.03	-0.05, -0.02	<0.001

B, unstandardized beta coefficient; BMI, body mass index; CI, confidence interval; FAS-III, Family Affluence Scale-III; Ref., reference; YAP-S, Youth Active Profile Spanish version.

Table S6. Unadjusted and adjusted associations between overall physical fitness and experiential avoidance among adolescents.

	Unadjusted model			Adjusted model		
**Predictor**	***B***	**95% CI**	***p*-value**	***B***	**95% CI**	***p*-value**
Overall physical fitness (per one SD)	-0.11	-0.28, 0.07	0.222	-0.04	-0.22, 0.15	0.696
FAS-III (per one point)				-0.45	-0.89, -0.02	0.043
Age (per one year)				0.23	-0.42, 0.88	0.485
Sex						
Boys				Ref.		
Girls				6.70	4.79, 8.61	<0.001
Energy intake (per 1000 kcal)				0.00	0.00, 0.00	0.184
YAP-S physical activity (per one point)				0.44	-0.96, 1.84	0.536
YAP-S sedentary behaviors (per one point)				1.59	0.02, 3.17	0.048
BMI (per one kg/m^2)				0.14	-0.07, 0.35	0.186
Overall sleep duration (per one hour)				-0.04	-0.05, -0.02	<0.001

B, unstandardized beta coefficient; BMI, body mass index; CI, confidence interval; FAS-III, Family Affluence Scale-III; Ref., reference; SD, standard deviation; YAP-S, Youth Active Profile Spanish version.
